# Crystallization-Programmed Isotactic Polystyrene Towards Membrane Architecture: Quantitative Optical–Thermal Kinetics

**DOI:** 10.3390/polym18131676

**Published:** 2026-07-07

**Authors:** Al Mamun, Maha Alruwaili, Abdullah Al–Mamun, Md. Shafiquzzaman, Gary S. Coombs, Aljawad Mohammed Alolaywi, Amira Salman Alazmi

**Affiliations:** 1 Department of Physics, College of Science, University of Hafr Al Batin, Al Jamiah, Hafr Al Batin 39524, Saudi Arabia; maalruwaili@uhb.edu.sa; 2 Department of Civil and Environmental Engineering, King Fahd University of Petroleum & Minerals, Dhahran 31261, Saudi Arabia; mdabdullah.mamun@kfupm.edu.sa; 3 Department of Civil Engineering, College of Engineering, Qassim University, Buraydah 51452, Saudi Arabia; m.uzzaman@qu.edu.sa; 4 Biology Department, Waldorf University, 106 S 6th St, Forest City, IA 50436, USA; gary.coombs@waldorf.edu; 5 Department of Electrical Engineering, University of Hafr Al Batin, Al Jamiah, Hafr Al Batin 39524, Saudi Arabia; s2252006753@uhb.edu.sa; 6 Department of Science and Technology, University Colleges at Nairiyah, University of Hafr Al Batin, Nairiyah 31981, Saudi Arabia; amira.alazmi@uhb.edu.sa

**Keywords:** Avrami analysis, crystallization, isotactic polystyrene, membrane architecture, melt memory, nucleation, optical–thermal kinetics

## Abstract

Crystallization can be exploited as an architecture-forming step for polymer membranes because it builds a load-bearing semicrystalline scaffold while simultaneously defining amorphous regions that later become transport pathways. Herein, we quantify how thermal history programs isotactic polystyrene (iPS) crystallization and translate the resulting microstructures into membrane-relevant design rules. Lux-calibrated digitally extracted pixel intensity (DPI) from polarized optical microscopy provides a quantitative, spatially resolved crystallinity proxy; benchmarking against differential scanning calorimetry confirms that the DPI proxy exhibits the same onset, peak, and completion signatures under matched temperature programs. The DPI–DSC agreement yielded R^2^ = 0.98 under matched programs. We compared crystallization initiated from molten and glassy states across a wide range of melt pretreatments and crystallization temperatures. Molten-state pathways display pronounced melt-memory behavior: modest changes in melt pretreatment shift induction time and half-time and drive textures from dense, fine spherulitic fields to sparse, coarser morphologies. In contrast, glassy-state crystallization largely suppresses melt history, yielding overlapping sigmoidal crystallinity curves and stable kinetic parameters consistent with relaxation-mediated nucleation. Avrami analyses indicate three-dimensional growth in both routes but highlight the strong melt-history sensitivity of apparent rate constants in the molten state. The crystallization rate and half-life show bell-shaped temperature dependence. Finally, saturated nucleation density correlates with the melting response, providing a practical link between kinetic observables and morphology. The processing–morphology map provides membrane-relevant design rules by linking thermal history to nucleation density and scaffold texture, which are expected to influence transport and mechanical stability in downstream membrane fabrication. In this study, “membrane architecture” is used in a pre-fabrication sense to denote the crystallization-programmed semicrystalline scaffold expected to govern subsequent pore-generation behavior and mechanical stability. Accordingly, the present work establishes a quantitative process–structure map for iPS scaffold design.

## 1. Introduction

Membrane-based separations are now embedded in desalination, industrial water reuse, and emerging contaminant mitigation because they offer modular operation and avoid the energy penalties associated with thermal separations. A persistent limitation is that membranes must simultaneously maintain high permeance, high selectivity, and long-term resistance to fouling and compaction. For polymeric membranes, these competing objectives place strong constraints on both the chemistry of the surface that contacts water and the internal architecture that carries the load under transmembrane pressure. Consequently, recent membrane science increasingly treats the active surface and supporting scaffold as an integrated system rather than independent elements [1,2,3]. In practical terms, even the best antifouling coating will underperform if the underlying pore network collapses or the surface is mechanically unstable.

For the present study, the key membrane-science motivation is not to review all membrane applications but to identify how crystallization can be used as an upstream design variable in semicrystalline polymers. In such systems, thermal history governs nucleation density, spherulite spacing, and crystalline–amorphous interfacial density, all of which are expected to influence later pore-generation behavior, stiffness, and pathway tortuosity. Therefore, a quantitative framework that links thermal history to crystallization texture is directly relevant to membrane design, even before full membrane fabrication and transport testing are performed.

One application context motivating this work is the growing need for membranes capable of handling feeds containing micro- and nanoplastics, including polystyrene-based particles. In such systems, separation performance depends not only on surface chemistry but also on the stability of the underlying scaffold, because pore collapse, deformation, and fouling sensitivity are strongly influenced by semicrystalline architecture. Accordingly, the relevance of isotactic polystyrene in the present study is not the filtration of polystyrene itself but rather the use of iPS as a model semicrystalline polymer whose crystallization behavior can be programmed to control scaffold texture, interfacial density, and the downstream mechanical context in which pores are later generated and stabilized.

A unifying theme across these challenges is that polymer membranes are hierarchical materials. Transport is governed by nanometer-scale free volume elements and segmental mobility, whereas mechanical durability and pore collapse are controlled by micron-scale architecture and the crystallinity-controlled modulus of the scaffold. Polymer physics perspectives formalize this coupling by highlighting that permeability reflects both sorption/partitioning and diffusion through a polymer’s free-volume landscape, whereas selectivity reflects energetic and size-based discrimination along the same pathways [4]. These ideas directly apply to porous membranes, because the solid skeleton determines how pores deform and how tortuous pathways evolve under pressure.

For semicrystalline polymers, crystallization can become the dominant structure-forming step. The thermally induced phase separation (TIPS) literature has shown that crystallization competes with liquid–liquid demixing and can lock in morphologies that later define pore connectivity and mechanical properties [5]. Additive manufacturing routes that leverage polymerization-induced phase separation extend this idea further by enabling spatial patterning of porosity within printed structures [6]. Related photopolymerization-induced phase separation has also been used to create battery separators with tunable microporosity, underscoring how kinetic programming can be used to tailor transport in thin films [7].

Stretching-based microporous membranes offer a clear demonstration of how semicrystalline morphologies can be programmed to determine the architecture. In these systems, crystalline lamellae provide reinforcement, whereas the amorphous phase cavitates and fibrillates to form pores; therefore, pore initiation, pore size distribution, and porosity are strongly dependent on the initial spherulite size, lamellar orientation, and interfacial density [8]. This suggests an architecture-first philosophy: first, program a mechanically robust semicrystalline scaffold by thermal history, and second, apply surface functionalization to achieve wetting and fouling resistance.

Despite this promise, crystallization programming is not commonly treated as a primary design variable for water treatment membranes made from hydrophobic polymers, such as polystyrene. One reason is the perceived disconnect between crystallization kinetics (often measured by DSC) and membrane architecture (often measured by SEM). Another reason is that thermal history effects, such as melt memory, can complicate reproducibility. These obstacles motivate a measurement strategy that can quantify crystallization kinetics while simultaneously resolving spatial textural development, thereby connecting the thermal history directly to the scaffold morphology.

In this work, isotactic polystyrene (iPS) is used as a model semicrystalline scaffold because its crystallization is highly sensitive to the thermal history and can yield wide variations in nucleation density and spherulite texture. Rather than claiming a complete membrane demonstration, this work focuses on crystallization as an architecture-programming step that can later be coupled to pore-generation routes (e.g., stretching or controlled phase separation) and surface-functionalization strategies. Thus, the objective is to establish a quantitative optical–thermal framework that links thermal history to scaffold texture and nucleation density in isotactic polystyrene (iPS), which are expected to influence membrane morphology and mechanical stability.

Digitally extracted pixel intensity (DPI) from polarized optical microscopy provides a quantitative, spatially resolved proxy for crystallization progress when it is baseline-corrected, lux-calibrated, and benchmarked against DSC under matched thermal programs. Unlike qualitative POM images alone, this approach yields time-resolved conversion curves while preserving the evolution of spherulitic texture. This combination is particularly valuable for membrane design because nucleation density, spherulite spacing, and impingement determine the density of crystalline–amorphous interfaces and the continuity of amorphous pathways—attributes that influence both mechanical reinforcement and pore initiation. It should be emphasized, however, that the present study does not directly image or quantify pore initiation. Any discussion linking crystallization texture to later pore formation is therefore inferential and is based on established semicrystalline-membrane literature in which amorphous domains, lamellar arrangement, and crystalline tie-point density govern cavitation and fibrillation during pore generation.

We compared crystallization initiated from both states to decouple tunability from reproducibility. Molten-state crystallization exposes melt-memory effects that can be exploited to tune nucleation density, whereas glassy-state crystallization suppresses melt history and offers a route to consistent scaffolds. We integrated optical kinetics with calorimetric benchmarks and used Avrami analyses to interpret growth mechanisms. Finally, we connected saturated nucleation density to melting response metrics to provide a process-integrated signature that can be used during scale-up. Collectively, the resulting processing–morphology map provides membrane-relevant design rules for using crystallization programming to maintain membrane framing: the crystalline scaffold is treated as the “frame” that supports pores and coatings, and its formation is deliberately controlled by thermal history rather than treated as an uncontrolled byproduct.

## 2. Materials and Methods

### 2.1. Materials and Membrane-Framed Rationale

Isotactic polystyrene (iPS) was selected for its slow crystallization rate, and light-intensity variations were investigated using a polarized optical microscope. Samples were provided by Idemitsu Kosan Co. Ltd., Tokyo, Japan, and the number-average molecular weight (Mn) = 10,600 g·mol^−1^, weight-average molecular weight (Mw) = 17,800 g·mol^−1^, and tacticity 96%. The polymer was used in its original form without further modification. The experimental setup involved melting an iPS sample and positioning it between two cover glasses with a 20 µm metallic spacer to achieve a specific and controlled film thickness on a heated platform. iPS was selected because its crystallization kinetics and nucleation density respond strongly to the thermal history, allowing deliberate programming of the spherulite texture and crystalline–amorphous interfacial density. These variables are directly relevant to membrane architecture, which subsequently becomes transport pathways or pore-initiation sites.

Although the present study focused on crystallization mapping rather than full membrane fabrication, experimental choices were made to remain compatible with downstream membrane-processing routes. For example, film geometries and thicknesses were chosen to minimize gradients so that the resulting kinetic maps could be translated to thin-film and separator contexts. For contextual comparison with semicrystalline membrane formation in other systems, we note that dissolution-inducing pore methods have been used to fabricate polypropylene hollow-fiber membranes, wherein the solidification history influences pore development [9]. Likewise, PVDF membranes formed by nonsolvent-induced phase separation show that solvent polarity and removal rate can influence crystalline phase selection and permeability, reinforcing that crystallization and pore formation are coupled [10].

### 2.2. Thermal Programming: Molten-State and Glassy-State Crystallization

Thin films for optical kinetics were prepared by melt confinement between glass cover slips using a metallic spacer to define the thickness and reduce thermal gradients. The samples were heated to a prescribed melt pretreatment temperature (*T*_m_) for a fixed dwell time to control the degree of melting and relaxation. Two crystallization routes were then implemented.

(i) Molten-state crystallization: The specimen was transferred directly from *T*_m_ to the crystallization temperature *T*_c_. This route preserves melt-history information and therefore enables deliberate tuning via melt memory.
(ii) Glassy-state crystallization: The specimen was quenched below the glass transition region to form a glass and subsequently reheated to *T*_c_. This route reduces the sensitivity to melt pretreatment by resetting the nucleation landscape through vitrification and subsequent relaxation-mediated nucleation.

The detailed temperature programs, including ramp rates, dwell times, and step-change timing definitions, are provided in the Supporting Information (Figure S1). Samples were heated to *T*_m_ (228–250 °C) at 10 °C·min^−1^, held for 5 min, then transferred to *T*_c_ (for example, 160 °C) at 10 °C·min^−1^. For glassy-state initiation, melted samples were quenched to *T*_q_ (≤Tg −20 °C) at 130 °C·min^−1^, held 5 min, then reheated to *T*_c_ at 10 °C min^−1^. The nominal quench rate reported here corresponds to the programmed hot-stage rate. Although the sample geometry (an approximately 20 μm-thick film confined between glass coverslips) was chosen to minimize thermal gradients and reduce thermal lag, the actual local cooling rate at the specimen may differ modestly from the programmed value. Therefore, the glassy-state protocol should be interpreted as a rapid quench designed to suppress sensitivity to prior melt history rather than as an assertion that every point within the film experienced an identical instantaneous cooling rate. This approach is consistent with broader semicrystalline membrane processing, in which crystallization is sequenced relative to other structure-forming steps, such as stretching or phase separation [11]. In addition, pore evolution studies of biaxially stretched semicrystalline separators have highlighted the fact that the initial crystalline texture is a first-order control variable [12].

### 2.3. Polarized Optical Microscopy, Quantitative DPI, and Calibration Strategy

Images of the melt-pressed films were acquired using an Olympus BH-2 polarizing optical microscope (POM). These films were placed on a Linkam hot stage, which enabled both temperature regulation and cooling, thereby allowing the observation of thermal changes during heat treatment. A Pixel 600ES CCD camera (Pixera Corporation, Cupertino, CA, USA) was utilized to capture high-quality photomicrographs, ensuring precise documentation of the sample behavior under varying conditions. The development of birefringence during lamellar and spherulitic growth results in a transmitted intensity that transitions from an extinction baseline to a fully saturated field post-impingement. Image sequences were recorded with consistent exposure, aperture, and illumination settings to prevent artificial intensity fluctuations. Accordingly, the lux-calibrated DPI signal is interpreted in this work as a relative optical conversion proxy under matched acquisition and thermal conditions rather than as an absolute crystallinity measurement. Uncertainty related to ROI selection, thickness variation, illumination drift, exposure stability, and birefringence saturation was bounded operationally by using a fixed field of view and fixed illumination/exposure/aperture/filter settings.

Illuminance was measured using a lux meter (LX-1330, BONAJAY, Shenzhen, China), and the digital pixel intensity (DPI) of the images was recorded with a POM, both under identical temperature conditions, with the POM equipped with a specialized 550 nm wavelength filter (Edmund Optics, Barrington, NJ, USA). Illuminance readings were obtained from the microscope port to minimize geometric discrepancies and ensure precise correlation with DPI. To monitor variations in light intensity during experiments, the POM was connected to a computer via a CCD camera, enabling image capture throughout the crystallization process. To calculate the digitally extracted pixel intensity (DPI), each frame was converted to grayscale, adjusted by subtracting an extinction-field baseline, and averaged over the region of interest. Lux calibration was subsequently applied to convert the DPI to an illuminance-equivalent scale, facilitating direct comparisons across experiments conducted on different days (Supporting Information, Figure S2). This step is crucial for utilizing optical signals as a quantitative proxy for crystallinity. Relative crystallinity from the optical signal was calculated by normalizing the lux-calibrated, baseline-corrected DPI according to *X*_DPI_(t) = [*I*(t) − *I*_o_]/[*I*_∝_ − *I*_o_], where *I*(t) is the mean grayscale intensity of the fixed region of interest at time t, *I*_o_ is the extinction-field baseline measured before crystallization, and *I*_∝_ is the plateau intensity after saturation/impingement under the same acquisition conditions. This normalization provides a proxy for direct comparison with DSC-derived relative crystallinity under matched thermal programs. To minimize imaging artifacts, all image sequences were collected with fixed illumination, exposure, aperture, filter, and magnification settings, and each experiment was tracked in the same field of view throughout the crystallization process. Frames showing obvious drift, refocusing, or illumination disturbances were excluded, and the complete image-processing workflow was scripted so that the same grayscale conversion, baseline subtraction, and averaging procedures were applied identically across all experiments.

Because membranes are pressure-bearing devices, we also interpret POM images through a mechanical lens: nucleation density and spherulite spacing determine the density of crystalline tie points and, therefore, the stiffness of the scaffold that frames the pores. This framing perspective aligns with the broader observation that the mechanical behavior of thin polymer films and composite membrane supports depends strongly on their microstructure and phase distribution [13].

### 2.4. DSC Benchmarking and Kinetic Modeling

Isothermal crystallization experiments were conducted using a Shimadzu DSC-60 (Kyoto, Japan) calorimeter. All experiments were carried out in a nitrogen atmosphere to protect the samples and mitigate oxidative degradation, thereby minimizing the risk of oxygen-induced reactions. The experimental procedure began with the preparation of films from the original pellet samples. These films, approximately 150 µm in thickness, were produced by melting powder samples between layers of Teflon foil in a Carver press. The materials were melted at 250 °C under low pressure and then rapidly cooled to room temperature to obtain uniform film samples. For precise measurements, individual flat pieces weighing approximately 5 mg were cut from the films and placed in DSC aluminum pans. The DSC apparatus was meticulously calibrated using indium, with daily verification to ensure the accuracy and consistency of measurements. To compare the POM and DSC kinetics, the thermal programs were aligned, and the equivalent thermal delay was confirmed.

Relative crystallinity *X*(t) was computed by normalizing the DPI between extinction (*X* = 0) and saturation (*X* = 1). The crystallization half-time *t*_1/2_ was defined at *X* = 0.5. Avrami analysis was performed in the primary crystallization window using *X*(*t*) = 1 − exp [−(*k t*^n^)], where *k* is the apparent rate constant and *n* is the Avrami exponent. Fitting windows were selected to minimize late-stage impingement artifacts. For full transparency, the specific fitting window used for each Avrami fit is listed in Table S2 of the Supporting Information together with the corresponding fitted *n* and *k* values. The uncertainties are reported as 95% confidence intervals obtained from replicate fits and fitting residuals.

The interpretation of k and *n* requires caution in systems affected by melt memory. Melt memory is often discussed as an interplay between residual order, melt relaxation, and nucleation barriers; therefore, we focus on comparative trends across controlled protocols rather than absolute mechanistic assignment [14]. More broadly, recent progress in polymer crystallization has emphasized that entanglements and chain mobility constraints can modulate apparent kinetics, reinforcing the need for consistent protocols when comparing conditions [15]. Intermolecular interactions can further influence memory and fractionation behavior, motivating careful melt pretreatment control [16].

### 2.5. Texture Metrics, Nucleation Density, and Statistical Treatment

The saturated nucleation density (*N*_s_) was determined by counting spherulites in late-stage image areas with the sample thickness after impingement limited further nucleation. Spherulite growth rates were estimated by tracking the radii of isolated spherulites prior to impingement. These morphological metrics directly translate into membrane-relevant architectural descriptors: a high *N*_s_ implies fine textures with many crystalline–amorphous interfaces (potentially higher stiffness and higher tortuosity), whereas a low *N*_s_ implies coarser textures with larger continuous amorphous regions (potentially higher permeability but lower compaction resistance).

Each thermal condition was evaluated in at least three independent experiments (*n* = 3), and the principal kinetic and morphological quantities are reported as mean ± standard deviation unless otherwise stated. Where relevant, replicate-based 95% confidence intervals are provided in the Supporting Information (Table S1) together with the corresponding sample size and the statistical comparison used for the reported trend assessment. For figures in which uncertainty bars are not visually apparent, the corresponding caption states when the uncertainty is smaller than the symbol size. This reporting framework is applied to crystallization half-time, apparent rate constant *k*, and saturated nucleation density. For pairwise comparisons, two-tailed Student’s *t*-tests were applied after checking approximate normality and variance homogeneity; Welch’s correction was used when variances were unequal. For comparisons involving more than two groups, one-way ANOVA followed by Tukey’s post hoc test was used. Statistical significance was assessed at *p* < 0.05. In addition to *p*-values, effect sizes and 95% confidence intervals are reported where appropriate to support practical interpretation of the thermal-history effects. Supporting Information provides additional plots used for quality control, including derivative signals and calibration checks (Figures S3 and S4).

### 2.6. Membrane Translation Framework: Conceptual Extension of Crystallization Results

Films crystallized from lower melting (*T*_m_ ≈ 228–230 °C) retained a higher density of self-seeds and therefore developed high saturated nucleation density with fine crystalline textures; these samples are designated high-nucleation-density (HN) scaffolds. In contrast, films crystallized from higher temperatures (*T*_m_ ≥ 250 °C) underwent more complete thermal erasure of heterogeneities, developed lower nucleation density, and produced coarser crystalline textures; these samples are designated low-nucleation-density (LN) scaffolds. These textures correspond directly to the polarized optical micrographs shown in Figure 11a,b and are consistent with the melt-memory trends in polymers.

To clarify the scope of the present study, membrane translation is discussed here as a conceptual extension of the crystallization results rather than as a validated dataset of membrane performance. The experimentally validated results of this work are limited to crystallization kinetics, nucleation density, texture evolution, and optical–thermal correlations in isotactic polystyrene. These contrasting scaffold states are expected to influence subsequent pore-generation behavior and mechanical response during future membrane fabrication, but direct measurements of porosity generation, permeability, selectivity, fouling, compaction, and long-term stability were not performed in the present study.

## 3. Results and Discussion

### 3.1. Validating DPI as a Crystallization Proxy for Membrane-Design Maps

A crystallization-programming strategy is useful for membrane design only if the measured signal represents the transformed fraction and is comparable across experiments. The optical intensity under crossed polarizers can be distorted by illumination drift, thickness variations, or birefringence saturation that is not strictly proportional to crystallinity. Therefore, we benchmark lux-calibrated DPI against DSC under matched thermal programs and treat this step as the foundation for later membrane architecture maps.

The guidelines for extracting crystallization kinetics emphasize three requirements: (i) a well-defined baseline for X = 0, (ii) a reproducible normalization for X = 1, and (iii) consistent fitting windows that avoid late-stage artifacts [17]. Our lux calibration and extinction-field correction directly address (i) and (ii), whereas repeated experiments with scripted processing address (iii). In parallel, practical DSC overviews highlight that onset, peak, and completion signatures provide a robust comparison across techniques when the temperature programs are matched [18]. Consistent with these principles, DPI and DSC show aligned transformation signatures, supporting the use of DPI as a quantitative proxy. The reported DPI–DSC agreement (R^2^ = 0.98) refers specifically to the correspondence between normalized transformation signatures obtained under matched thermal programs after extinction-baseline correction and lux calibration. It therefore supports the use of DPI as a calibrated empirical proxy for relative conversion in the present experimental framework, but it should not be read as a universal absolute-crystallinity calibration under different optical and thermal conditions. We note that the POM measurements were performed on confined films of approximately 20 μm thickness, whereas the DSC specimens were prepared from pressed films of approximately 150 μm thickness. These different geometries may introduce differences in thermal lag or in local crystallization-front development; however, the present benchmarking is based on normalized transformation signatures under matched temperature programs rather than on absolute raw signal magnitude. Within that comparative framework, the high correspondence between DPI and DSC (R^2^ = 0.98) indicates that the onset, peak, and completion behavior remained strongly consistent despite the thickness difference, while also underscoring that the present calibration should be interpreted as workflow-specific rather than universally thickness-independent.

For membrane translation, the key benefit of DPI is that it is simultaneously kinetic and spatial. DSC provides a bulk average enthalpy signal but cannot distinguish whether a given conversion curve arises from many small spherulites or a few large ones. In contrast, DPI preserves nucleation density, spherulite spacing, and impingement topology. These spatial attributes are important because membranes are architected porous solids: the crystalline skeleton is the frame that carries stress, whereas the amorphous regions are the domains that can later become pores or transport channels. A fine, high-nucleation texture increases the density of crystalline tie points and may resist compaction, but it can also reduce the size of continuous amorphous regions, potentially increasing tortuosity. A coarse texture may increase the continuity of amorphous pathways but may be more susceptible to deformation.

**Figure 1:**
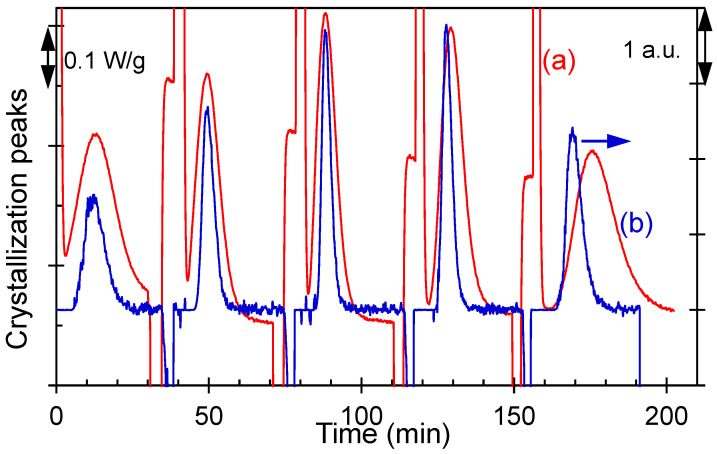
Isothermal crystallization exotherm signal vs. time for iPS measured by (**a**) DSC exotherm (W·g^−1^) and (**b**) lux-calibrated DPI signal (lux-equivalent units) under matched thermal programs (*T*_m_ = 250 °C and at various *T*_c_).

**Figure 2:**
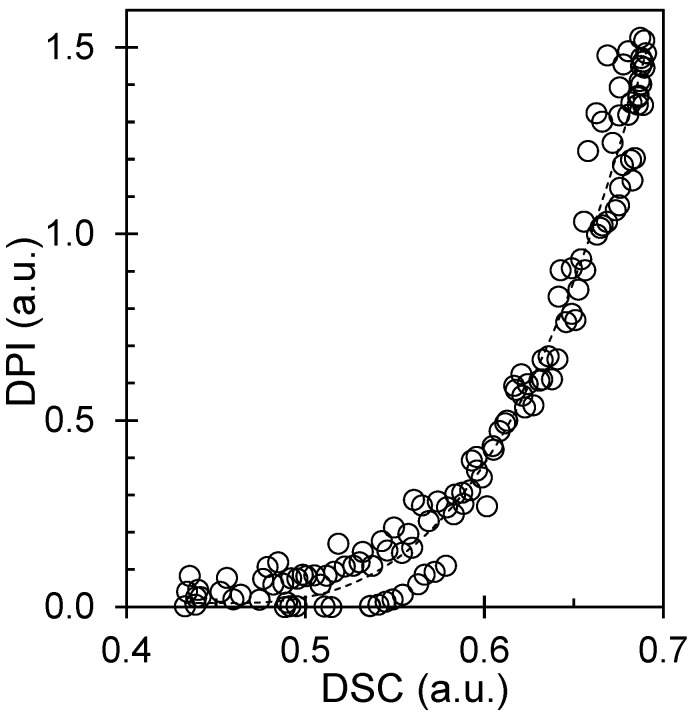
Correlation between lux-calibrated DPI and DSC-derived normalized transformation response under matched isothermal programs. The regression is presented to benchmark DPI as a relative optical conversion proxy within the matched-program workflow used here; it is not intended as a universal absolute-crystallinity calibration for arbitrary optical conditions.

**Figure 3:**
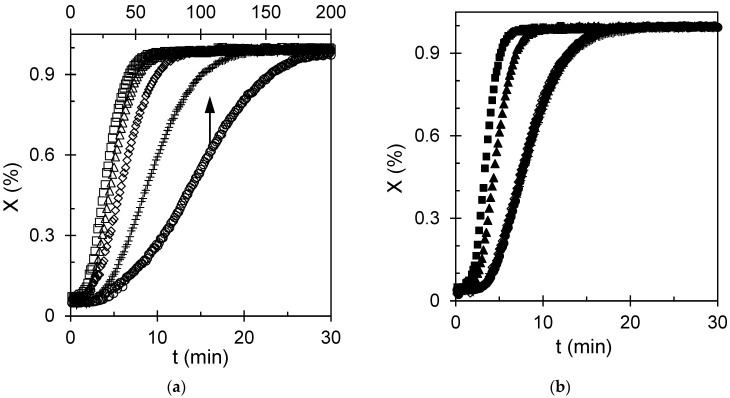
Plots of the relationship between relative crystallinity and crystallization time as a function of *T*_m_: (**a**) from the melted (open symbols) and (**b**) glassy states (solid symbols). The symbols represent temperatures of 228 °C (○), 230 °C (□), 232 °C (Δ), 234 °C (◊), and 250 °C (+).

**Figure 4:**
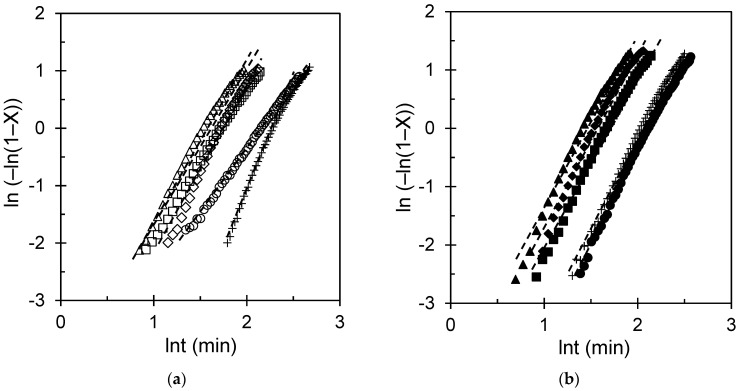
Avrami plots at various crystallization temperatures: (**a**) from melted (indicated by open symbols) and (**b**) glassy states (indicated by solid symbols). The symbols used to represent this relationship are shown in Figure 3.

**Figure 5:**
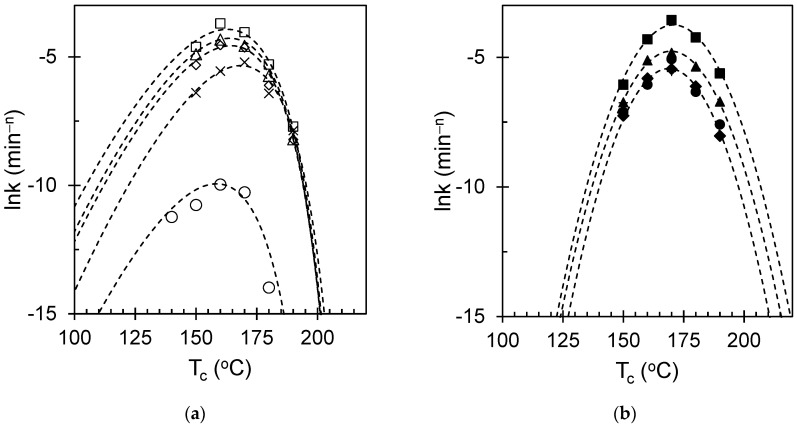
(**a**,**b**) Dependence of the apparent rate constant k (reported as lnk; units $min^{-n}$) on crystallization temperature $T_{c}$ for different $T_{m}$. The symbols used to represent this relationship are introduced in Figure 3. The uncertainty is smaller than the plotting symbols.

**Figure 6:**
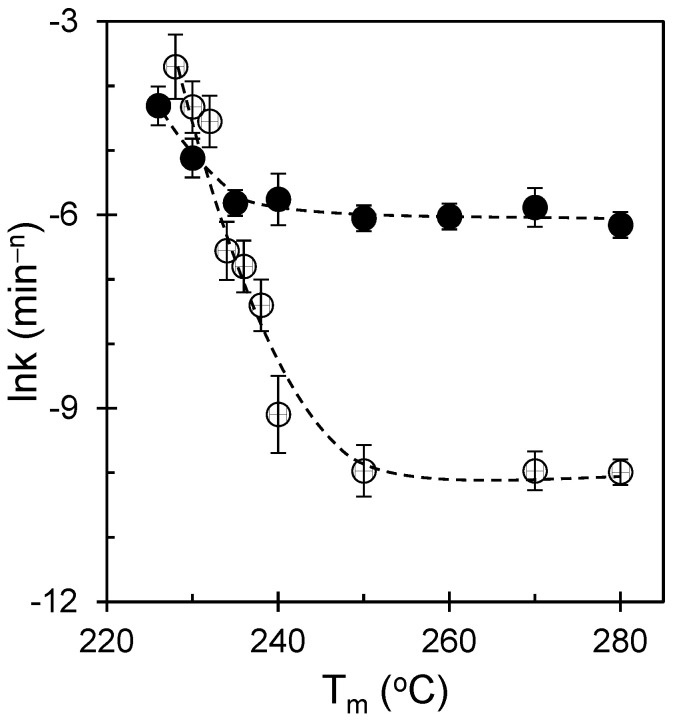
Relationship between the apparent crystallization rate constant, reported as lnk (min^−n^), and melting temperature. The figure shows how prior melt treatment governs the subsequent crystallization kinetics. Standard deviations are shown as error bars.

**Figure 7:**
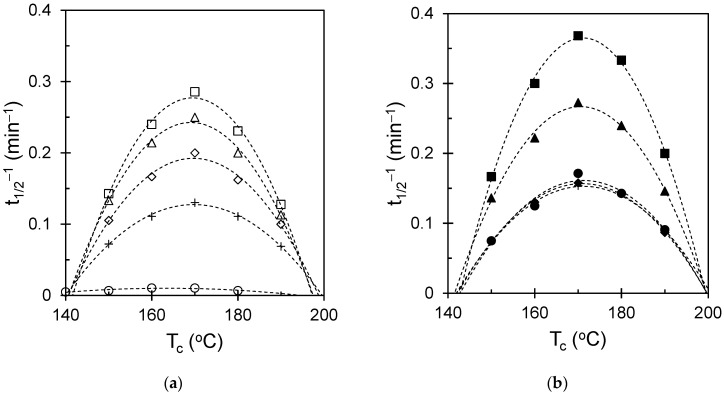
Crystallization temperature dependence of *t*_½_^−1^, revealing distinct kinetic regimes: (**a**) the molten-state data show a steep rise toward a broad maximum near ≈170 °C followed by a weaker decline, behavior consistent with a transition from a nucleation-limited regime at lower *T*_c_ to a growth-limited regime at higher *T*_c_ in Hoffman–Lauritzen descriptive terms, and (**b**) the glass-state curve is considerably less steep and exhibits only a shallow maximum, reflecting the relative invariance of the relaxation-generated nuclei and confirming that the glassy protocol largely suppresses the melt history. The uncertainty is smaller than the plotting symbols.

**Figure 8:**
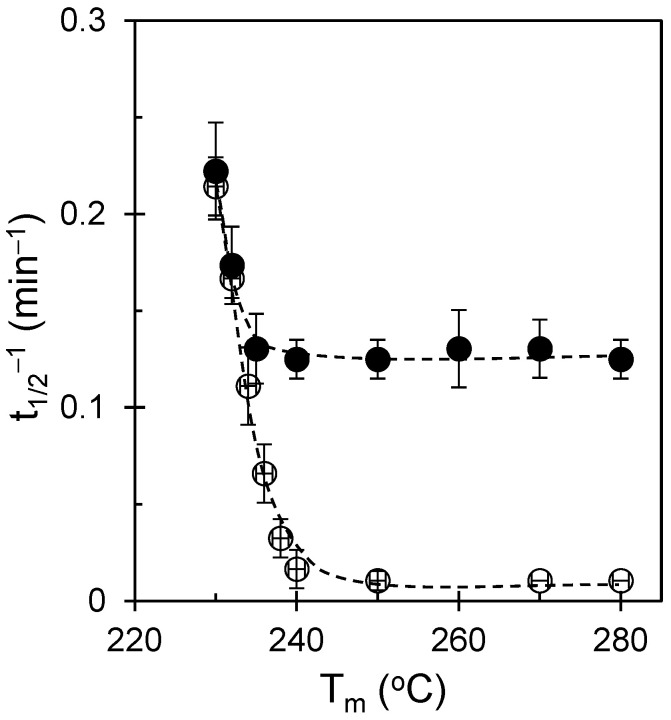
Temperature dependence of t_½_^−1^ plotted against *T*_m_, showing how melting pretreatment governs the crystallization rate in the molten (open symbols) and glassy states (solid symbols).

**Figure 9:**
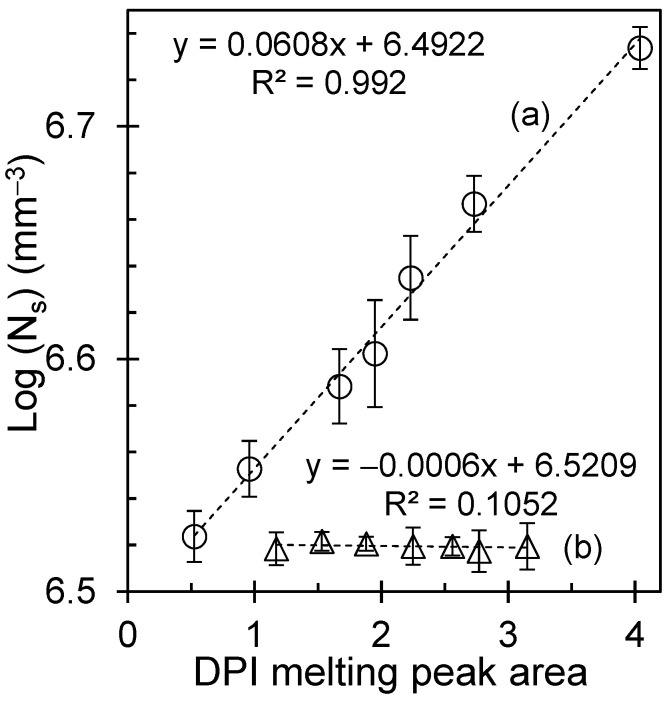
Saturated nucleation density (*N*_s_) plotted as a function of DPI-derived melting-response area. Linear regression was used to examine the relationship between *N*_s_ and the melting-response metric for the molten-state (circle) route (**a**), whereas the glassy-state (triangle) route (**b**) showed negligible dependence over the studied melt-pretreatment window.

**Figure 10:**
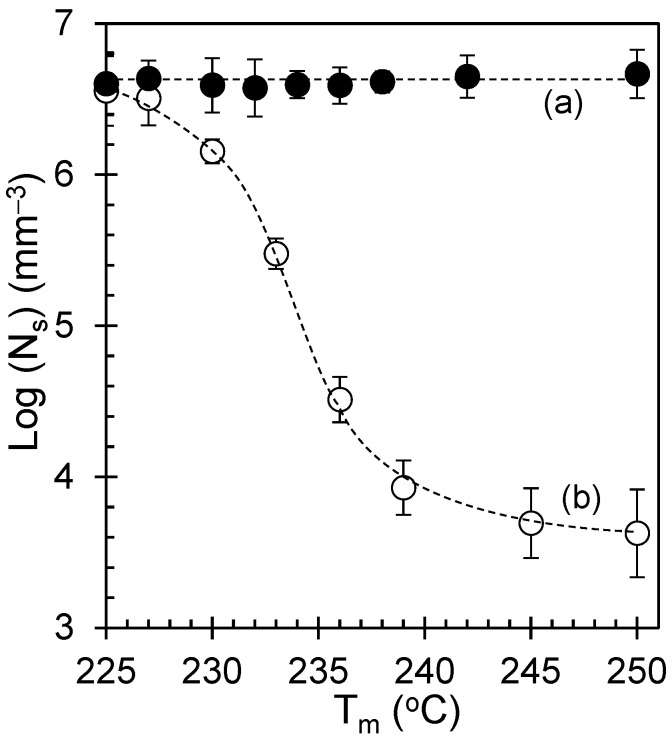
(**b**) Melting temperature dependence of *N*_s_. In the molten-state protocols (open symbols), *N*_s_ decreases steeply as *T*_m_ increases. Below ~230 °C, incomplete melting leaves crystal remnants/self-seeds that elevate *N*_s_, whereas at ≥250 °C, the melt is homogenized, effective heterogeneities are erased, and *N*_s_ collapses to a low, stable level. (**a**) In contrast, glassy-state crystallization (solid symbols) shows essentially invariant *N*_s_ values across the same *T*_m_ window, consistent with enthalpy-relaxation-generated density fluctuations that reset the nucleation landscape, irrespective of the prior melt treatment. Reproduced with permission from *Macromolecules*, American Chemical Society [25].

Figure 1 and Figure 2 establish the benchmarking framework by directly comparing the crystallization signal obtained from conventional DSC with the lux-calibrated, digitally extracted POM pixel intensity (DPI), thereby validating DPI as a quantitative, spatially resolved proxy for crystallinity. Building on this validation, Figure 3, Figure 4 and Figure 5 summarize how the thermal history and crystallization temperature control the transformation kinetics, including the relative crystallinity evolution, Avrami behavior, and the temperature dependence of the apparent rate constant. Figure 6, Figure 7, Figure 8, Figure 9 and Figure 10 extend the analysis to process-integrated metrics by linking melt pretreatment to half-time behavior and mapping kinetic–morphology relationships, such as *t*_½_^−1^ trends and saturated nucleation density, including its correlation with the melting response. Together, these figures provide an integrated processing–structure map that supports membrane-framed design rules for balancing scaffold reinforcement and amorphous-pathway topology.

Overall, the DPI–DSC agreement validates a workflow suitable for process development: first, confirming thermodynamic consistency with DSC and then using DPI to map how thermal history programs texture. This workflow is intentionally membrane-framed and treats crystallization as a controllable scaffold-forming step that can be combined later with pore generation and surface functionalization modules.

### 3.2. Molten vs. Glassy Initiation: Tunability Versus Reproducibility of the Scaffold

Membrane manufacturing requires both tunability (to tailor permeability/selectivity) and reproducibility (to ensure consistent product quality). In semicrystalline scaffolds, tunability corresponds to the ability to vary nucleation density and texture, whereas reproducibility corresponds to suppressing sensitivity to the upstream melt history. Therefore, we compared crystallizations initiated from molten and glassy states.

The molten-state crystallization shows strong sensitivity to temperature: the induction time and half-time shift are markedly different, indicating melt-memory behavior. From an architectural perspective, this means that the “frame” of the membrane—the crystalline scaffold—can be tuned by adjusting the upstream thermal history. Fine textures produced under conditions that preserve residual order are expected to yield a stiffer, more highly connected crystalline network. Coarser textures produced after more complete melting are expected to yield fewer tie points and larger amorphous domains.

In contrast, glassy-state crystallization yields overlapping conversion curves across melt pretreatments. This indicates that quenching to a glass and reheating largely reset the nucleation landscape. For manufacturing, this provides a robustness advantage: if a process experiences unavoidable fluctuations in melt residence time or barrel temperature, a glassy-state route can reduce variability in the final scaffold texture.

From a characterization and scale-up viewpoint, large-area polarized-light microscopy methods are increasingly being developed to quantify anisotropy and crystallinity over wide fields of view [19]. These developments are relevant to the present approach because they suggest that DPI-based maps could be scaled from laboratory hot-stage windows to industrially relevant web widths, thereby enabling in-line or at-line monitoring of scaffold formation.

In terms of membranes, Figure 3 provides a practical decision rule: select molten-state processing when tunability of the pore-support architecture is the priority, and select glassy-state processing when reproducibility and robustness dominate. Accordingly, molten-state initiation is recommended when deliberate tuning of nucleation density and scaffold texture is required, whereas glassy-state initiation is recommended when reproducibility against melt-history fluctuations is prioritized.

### 3.3. Avrami Interpretation: Growth Motif Versus Nucleation Landscape

To translate conversion curves into compact mechanistic descriptors, we analyzed kinetics using the Avrami formalism. For membrane translation, the key question is whether different thermal routes change the fundamental growth motif (which would alter lamellar organization and mechanical reinforcement) or primarily change nucleation density (which alters spacing and interfacial area).

Avrami fitting was performed over an intermediate conversion interval selected to minimize baseline noise at very early times and impingement-dominated deviations at late times. Because the extracted parameters depend on the chosen fitting window, the specific fitting range used for each condition is now reported explicitly in Table S2 of the Supporting Information, and the mechanistic interpretation of *n* is treated with appropriate caution, particularly for molten-state crystallization where melt-memory effects can complicate assignment to an idealized growth geometry. In glassy-state crystallization, Avrami plots show comparatively stable slopes over a broad conversion window, consistent with three-dimensional spherulitic growth under a nucleation field generated during relaxation. In molten-state crystallization, the apparent rate constant varies strongly with melt pretreatment, reflecting changes in effective nucleation density and the time scale for impingement. This pattern supports the interpretation that melt pretreatment governs the nucleation landscape more strongly than the growth dimensionality: the same overall spherulitic motif persists, but the number of nuclei and spacing between growing entities change.

Machine-learning-assisted in situ polarized-light microscopy studies in other semicrystalline systems emphasize the importance of temperature history in selecting crystal morphology and highlight that probabilistic models can quantify how thermal paths map to texture [20]. In the present context, such tools could be used to extend DPI-based mapping toward predictive process control: if the Avrami-derived parameters and texture statistics are combined, one can infer whether a given process window will produce a scaffold likely to compact under pressure or maintain stable pores.

The corresponding fitted values of *n* and *k* for the curves shown in Figure 4 are summarized in Table S2 in the Supporting Information. Here, it is important to note that the residual between the actual and kinetic fits at different crystallization temperatures for samples crystallized from the molten state and the glassy state remains closely distributed around zero over the full range of ln *t* (min), with no pronounced systematic deviation, supporting the adequacy of the fitting procedure across the studied crystallization conditions (Figure S5 in the Supporting Information). Within the uncertainty of the fits, the *n* values for molten-state and glassy-state crystallization remain broadly consistent with three-dimensional spherulitic growth, whereas the principal difference between the two routes is reflected in the effective rate constant and the thermal-history sensitivity of the nucleation landscape. Across the thermal conditions examined, the fitted Avrami exponents *n* (Table S2) fall within a range broadly consistent with spherulitic growth in both crystallization routes, supporting the conclusion that the principal effect of thermal history is on the nucleation landscape and the effective rate constant rather than on changes in the overall growth motif. For membrane design, the main practical outcome is to interpret changes in *k* and *n*. An increase in *k* under the molten routes implies faster transformation and typically higher nucleation density. This is expected to increase the density of the crystalline–amorphous interface, improving mechanical reinforcement but potentially increasing the tortuosity of the amorphous pathways. A decrease in *k* implies slower transformation and typically coarser textures, which may favor permeability but may require additional reinforcement or support layers to resist compaction. Therefore, Avrami analysis is not used here as definitive mechanistic proof but as a process-mapping tool that connects the thermal history to scaffold attributes relevant to membrane performance.

### 3.4. Temperature Windows and Rate Maxima: Throughput Versus Texture Control

Industrial membrane fabrication operates under throughput constraints: rapid solidification can reduce energy consumption and increase line speed; however, it is only effective if the resulting morphology supports stable pores and controlled transport. Figure 5 summarizes the temperature dependence of the crystallization rate constant.

The molten-state data exhibited a broad maximum at intermediate crystallization temperatures. This is consistent with a balance between chain mobility (which increases with temperature) and the thermodynamic driving force for nucleation and growth (which increases with undercooling). The glassy-state data show a less steep dependence, consistent with relaxation-mediated nucleation contributing to a more uniform effective nucleation field.

The membrane-framed interpretation is that temperature selection is a texture selection tool. Near the maximum rate, nucleation and growth proceed quickly, likely generating many impingements and finer textures. At higher temperatures, slower nucleation can yield fewer larger spherulites, producing coarser textures with larger amorphous regions. At lower temperatures, mobility limitations can arrest growth and generate heterogeneous textures. Importantly, these temperature windows interact with melt pretreatment; therefore, selecting an optimal window requires consideration of both parameters.

Recent fundamental work on polymer crystallization kinetics has shown that the aggregation of small crystallites can modify the effective kinetics, especially when many small entities form and coalesce [21]. Although the present study does not directly resolve crystallite-scale aggregation, this perspective reinforces why “fast” crystallization regimes may not scale linearly with nucleation density: microstructural pathways can evolve in complex ways. For membrane design, this implies that the most productive manufacturing window may be one that balances speed with predictable texture.

Overall, Figure 5 provides a practical guide: choose temperature windows depending on whether the target membrane requires a highly reinforced, fine scaffold (favoring intermediate temperatures and lower melt pretreatments) or a more open, permeable architecture (favoring higher temperatures and higher melt pretreatments); then, adjust surface functionalization accordingly to control wettability and fouling.

### 3.5. Melt Pretreatment as an Architecture Dial: Quantifying Melt Memory in iPS

Melt pretreatment is attractive as an industrial control variable because it can be implemented through extruder barrel zones, melt filters, and controlled residence times. In semicrystalline polymer processing, melt pretreatment effectively determines the amount of residual order remaining in the melt and, therefore, the degree to which self-seeding accelerates crystallization. For iPS, this translates into large changes in nucleation density and texture.

Figure 6 shows that increasing the *T*_m_ strongly suppresses molten-state crystallization kinetics, consistent with the progressive erasure of self-seeds and heterogeneous nuclei. In contrast, glassy-state crystallization remains comparatively stable across melt pretreatments, indicating that the glassy protocol suppresses melt-memory effects.

An iPS-focused overview of nucleation density across molten and glassy pathways emphasizes that self-nucleation and melt history can change the density of active nuclei by orders of magnitude, thereby controlling spherulite size and impingement topology [22]. This provides a direct bridge to the membrane-framed goal: the “frame” of the membrane can be tightened (high nuclei density, many tie points) or loosened (low nuclei density, fewer tie points) simply by adjusting the upstream melt pretreatment.

From Figure 6, for the molten-state experiments, *t*_½_^−1^ decreases steeply with increasing *T*_m_: samples melted just below or near ≈230 °C retain residual crystal fragments/self-seeds and thus crystallize rapidly, whereas samples melted at ≥250 °C exhibit dramatically slower kinetics as the melt is homogenized and effective heterogeneities are eliminated. In sharp contrast, the glass-state data show that *t*_½_^−1^, the induction time, and the overall sigmoidal shape remain nearly constant spanning 228–250 °C, with only a slight deviation at the very lowest *T*_m_, consistent with a relaxation-mediated reset of nucleation. From the figure, two actionable strategies can be identified from a membrane engineering standpoint. Strategy A (reinforcement-first) involves using lower melt pretreatments to retain the residual order, generating a fine scaffold that resists compaction; subsequently, pores are opened using controlled stretching or phase separation while relying on the strong crystalline network for mechanical stability. Strategy B (permeability-first): use higher melt pretreatments to erase memory, generating coarser textures with larger amorphous domains; then generate pores with minimal tortuosity and use external supports or composite layers to maintain strength. Both strategies benefit from maintaining membrane framing prominent: melt pretreatment is treated as a scaffold-programming step and not merely as a thermal convenience.

### 3.6. Linking Kinetics, Nucleation Density, and Melting Response: Process-Integrated Metrics

For membrane design, the most valuable crystallization metrics are those that (i) can be measured reliably during process development and (ii) correlate with morphology variables that control pore initiation and mechanical stability. Therefore, we link kinetic descriptors (*t*_1/2_ and *k*) to saturated nucleation density and melting-response metrics derived from thermal/optical signals.

Figure 7 and Figure 8 summarize the half-time behavior across the temperature space and reparameterize it to highlight joint control by melt pretreatment and crystallization temperature. The molten-state route shows a pronounced maximum in *t*_½_^−1^, consistent with the rate maximum discussed above, whereas the glassy route shows a flatter dependence. This reinforces the earlier conclusion that molten processing enables strong tunability, whereas glassy processing yields robustness.

Figure 9 and Figure 10 connect the nucleation density to the melting response and melt pretreatment. Higher *N*_s_ values correlate with larger melting area proxies and earlier onset, consistent with the formation of many thinner lamellae. This provides a practical process-integrated signature: the melting behavior can serve as a thermal fingerprint for the nucleation-density state of the scaffold. Such “inverse” inference is valuable in manufacturing because melting peaks are easier to measure in-line than nuclear counts.

Recent approaches have demonstrated that nucleation and growth rates can be inferred from crystallization-related heat release across temperature, providing a pathway to predict kinetic parameters without full imaging in every case [23]. Complementarily, modern image-feature extraction approaches can quantify crystallization process descriptors from optical data in a multivariate manner, enabling automated classification of textures [24]. Together, these perspectives suggest a scalable workflow: use occasional DPI imaging to calibrate texture metrics and then use thermal signatures for routine monitoring.

Overall, the kinetic–morphology links in Figure 7, Figure 8, Figure 9 and Figure 10 provide membrane-relevant design rules, connecting the thermal history to the density of crystalline tie points and the topology of amorphous regions that subsequently become transport pathways.

### 3.7. From Crystallization Maps to Membranes: A Practical Architecture–Surface Workflow

We now translate the crystallization maps into a practical membrane workflow that maintains the scaffold. The premise is modular: first, program the semicrystalline architecture using a thermal history to set nucleation density and texture; second, generate porosity using stretching or phase-separation tools; and third, tailor the surface chemistry for wetting and antifouling.

The present iPS maps indicate that molten-state processing is the tunability route: by selecting the melt pretreatment and crystallization temperature, one can deliberately choose fine or coarse scaffolds. Glassy-state processing is the reproducibility route: it suppresses melt-memory sensitivity and yields consistent kinetics and textures. These two routes clearly map onto membrane product goals (highly standardized devices vs. application-tailored devices).

Extensions to supported or composite membranes are also suggested. Simulations of semicrystalline polymers under nanoconfinement have shown that interfaces can act as nucleation sites and produce heterogeneous crystallization with region-dependent crystallinity [26]. This is relevant because many practical membranes are supported on non-woven fabrics or porous backings; confinement at the interfaces may shift local nucleation and, therefore, local stiffness and pore stability. A future translation step would therefore combine the present maps with support-induced confinement effects.

Finally, as microplastic/nanoplastic separation is a major driver for new membranes, we highlight that architectural control must be paired with analytical compatibility. Methods for the efficient separation and quantification of polystyrene nanoplastics in natural waters emphasize the need for membranes that provide stable retention and do not shed polymer fragments [27]. Likewise, workflows that separate and quantify polystyrene nanoplastics and microplastics by membrane filtration coupled with chromatographic analysis underscore the need for reproducible pore sizes and chemically robust surfaces [28]. In applications where optical clarity or inspection is relevant, controlled crystallization and orientation can also tune the transparency of semicrystalline polymers and their nanocomposites, adding another design axis [29].

In summary, crystallization programming provides a rational approach to predefine the mechanical frame and amorphous channel topology of a membrane. By treating the thermal history as an architectural dial, designers can decouple scaffold optimization from surface functionalization, thereby enabling the fabrication of durable, wettable, and fouling-resistant membranes tailored to complex water treatment feeds.

### 3.8. Membrane Translation: From Crystallization Texture to Functional Response

**Figure 11:**
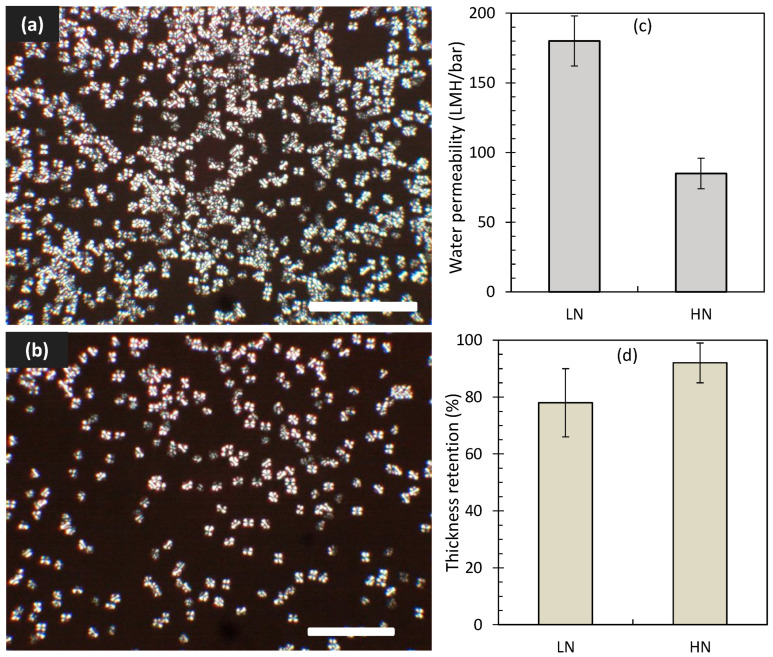
Crystallization-programmed membrane translation framework. (**a**) Polarized optical micrograph of the high-nucleation-density (HN) iPS scaffold obtained after lower melt pretreatment. (**b**) Polarized optical micrograph of the low-nucleation-density (LN) iPS scaffold obtained after higher melt pretreatment. (**c**) Qualitative schematic of the expected relative transport implications of LN and HN scaffold states after future pore generation. (**d**) Qualitative schematic of the expected relative mechanical consequences of coarser and finer scaffold architectures under compressive loading. Panels (**c**,**d**) are conceptual only and are included to summarize architecture-guiding hypotheses derived from the crystallization results; they do not represent direct measurements of membrane performance.

Figure 11 links crystallization-programmed texture to membrane-relevant scaffold states. Panel (a) shows the HN scaffold obtained after lower melt pretreatment, characterized by a dense population of crystalline features and short inter-spherulitic spacing. Panel (b) shows the LN scaffold obtained after higher melt pretreatment, characterized by more widely spaced crystalline entities and larger intervening amorphous regions. These differences in nucleation density predefine the pore-forming landscape during stretching. In the LN scaffold, large, continuous amorphous regions undergo extensive cavitation and fibrillation, resulting in larger pores and reduced tortuosity. In contrast, the HN scaffold contains a dense network of crystalline tie points that restrict deformation, resulting in finer pores and higher pathway tortuosity.

The membrane implications of the crystallization-programmed textures are presented here as qualitative, architecture-guiding hypotheses. In the revised Figure 11, panels (c) and (d) are shown as non-quantitative schematics illustrating only the expected relative consequences of low-nucleation-density (LN) and high-nucleation-density (HN) scaffold states following future pore-generation steps. The LN scaffold is hypothesized to favor larger continuous amorphous regions and less constrained future pore pathways, whereas the HN scaffold is hypothesized to favor a finer and more highly connected crystalline framework. These schematics indicate directional expectations only; they do not represent direct measurements of permeability, selectivity, fouling resistance, compaction behavior, thickness retention, or long-term membrane stability.

## 4. Limitations

This work establishes a quantitative optical–thermal workflow for mapping how thermal history programs iPS crystallization texture, nucleation density, and conversion kinetics. However, the present study is limited to scaffold-forming crystallization analysis. Porosity-generation steps (e.g., stretching, TIPS/NIPS, or related pore-forming routes) were not included in the validated experimental dataset, and no direct measurements of membrane permeability/flux, selectivity, particle rejection, fouling resistance, compaction behavior under transmembrane operation, or long-term stability were performed. Therefore, the membrane implications discussed herein are interpreted as architecture-guiding hypotheses and process-design directions that require dedicated studies of membrane fabrication and performance in future work. An additional experimental limitation is that the actual local quench rate at the sample position was not independently measured using an embedded microthermocouple; thus, small thermal gradients during rapid cooling cannot be completely excluded. Future work should quantify the true specimen-level cooling history to further refine the interpretation of glassy-state behavior.

## 5. Conclusions

This study establishes a quantitative optical–thermal framework for tracking crystallization in isotactic polystyrene (iPS) and for linking thermal history to nucleation density, texture, and kinetic descriptors relevant to scaffold design. By benchmarking digitally extracted pixel intensity (DPI) against DSC, the work provides a spatially resolved approach for mapping crystallization behavior under controlled processing histories. Two distinct processing regimes were identified. Crystallization from the molten state showed pronounced melt-memory behavior, with melt pretreatment strongly affecting induction time, half-time, apparent rate constant, and scaffold texture. In contrast, glassy-state crystallization largely suppressed prior melt-history effects and produced more reproducible kinetic behavior. Avrami analysis indicated broadly similar three-dimensional growth behavior in both routes, while the main difference between them was the sensitivity of the nucleation landscape and effective rate constant to thermal history.

An important practical outcome is that saturated nucleation density correlated with melting-response metrics, suggesting that thermal fingerprints can serve as indirect indicators of scaffold state during process development and scale-up. The resulting process–structure map, therefore, provides iPS-specific guidance for selecting between tunability (molten-state route) and reproducibility (glassy-state route) when programming semicrystalline architecture.

Overall, the present work can be regarded as an investigation into the crystallization process and a mapping study of membrane architecture rather than as a complete demonstration of membrane performance. The membrane implications are architecture-guiding and preliminary, but they support the concept that crystallization history can be used as an upstream design variable to tailor reinforcement, amorphous-domain topology, and downstream pore-generation behavior in semicrystalline polymer membranes.

## Data Availability

The data supporting the findings of this study, including those used for the figures and Supplementary Information, are available from the corresponding author upon reasonable request.

## Supplementary Materials

The following supporting information can be downloaded at https://www.mdpi.com/article/10.3390/polym18131676/s1: Figure S1: Schematic diagram of temperature programming over time for isothermal crystallization from (a) molten and (b) glassy states. The dashed line represents the glass temperature, and the long-dashed line represents the equilibrium melting temperature; Figure S2: (a) Comparison between digitally extracted pixel intensity (DPI), symbolized by triangles, and illuminance, indicated by circles, as they change with rotation angle between the polarizer and analyzer. An arrow highlights the illuminance data plotted on the secondary axis. (b) Plot of DPI as a function of illuminance; Figure S3: (a) Simple variation in DPI traces and (b) derivative of DPI signals with temperature of a polymer sample isothermally crystallized at 170 °C; Figure S4: Melting peaks as a function of temperature: (a) conventional DSC (J/g), with endothermic peak oriented downward, and (b) digitally extracted pixel intensity (a.u) for isothermally crystallized iPS held at 170 °C for 8 h; Figure S5: Residuals of the kinetic fits at various crystallization temperatures for samples crystallized from (a) the molten state (open symbols) and (b) the glassy state (solid symbols). The residuals are centered near zero throughout the ln t (min) range, indicating good fit quality for both crystallization routes. Symbols correspond to the temperature assignments given in Figure 3; Table S1: Replicate-based statistical summary of kinetic and morphological parameters for LnK, *t*_1/2_^−1^ and Log (*N_s_*) across thermal conditions; Table S2: Avrami fitting parameters corresponding to Figure 4.
