# Supplementary material for: Crystallization-Programmed Isotactic Polystyrene Towards Membrane Architecture: Quantitative Optical–Thermal Kinetics
Source: Polymers (Basel). 2026 Jul 7;18(13):1676. doi: 10.3390/polym18131676 (PMC13363971; doi:10.3390/polym18131676)
Supplement: Supplementary file 1 [file polymers-18-01676-s001.zip › polymers-4344245-supplementary.pdf]

Supplementary Information

Section Summaries

Temperature Programs: Detailed molten-state and glassy-state temperature programs and timing definitions used throughout the study (Figure S1).

Calibration and Quantitative Imaging: Lux calibration, extinction-baseline correction, and DPI extraction workflow used to compute relative crystallinity (Figure S2).

Auxiliary Analyses: Additional DPI traces, derivatives, and cross-checks used to validate kinetic assignments and reproducibility (Figures S3–S4).

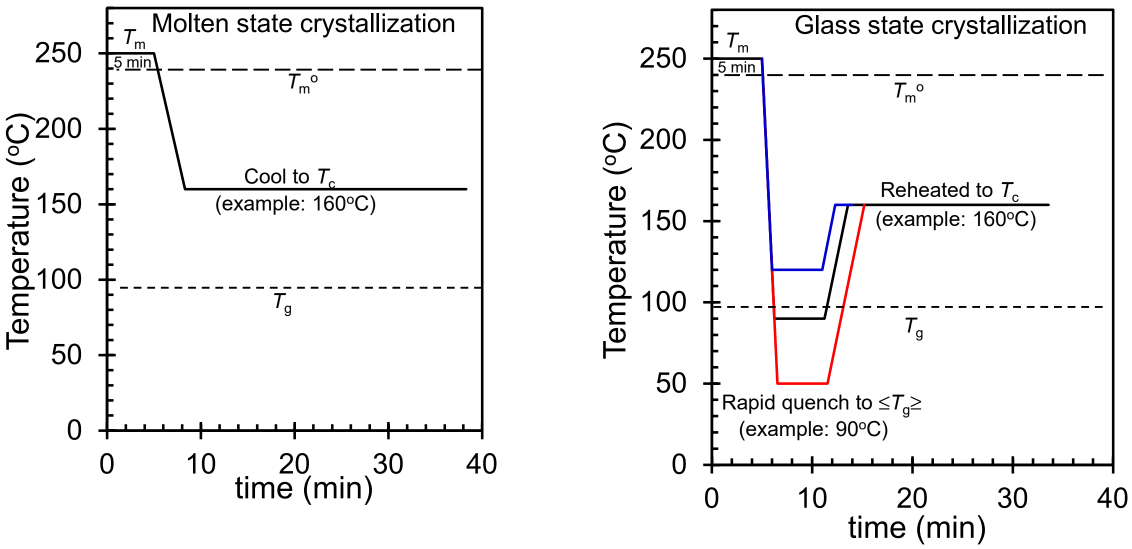

(**a**) (**b**)

Figure S1. Schematic diagram of temperature programming over time for isothermal crystallization from (a) molten and (b) glassy states. The dashed line represents the glass temperature, and the long-dashed line represents the equilibrium melting temperature.

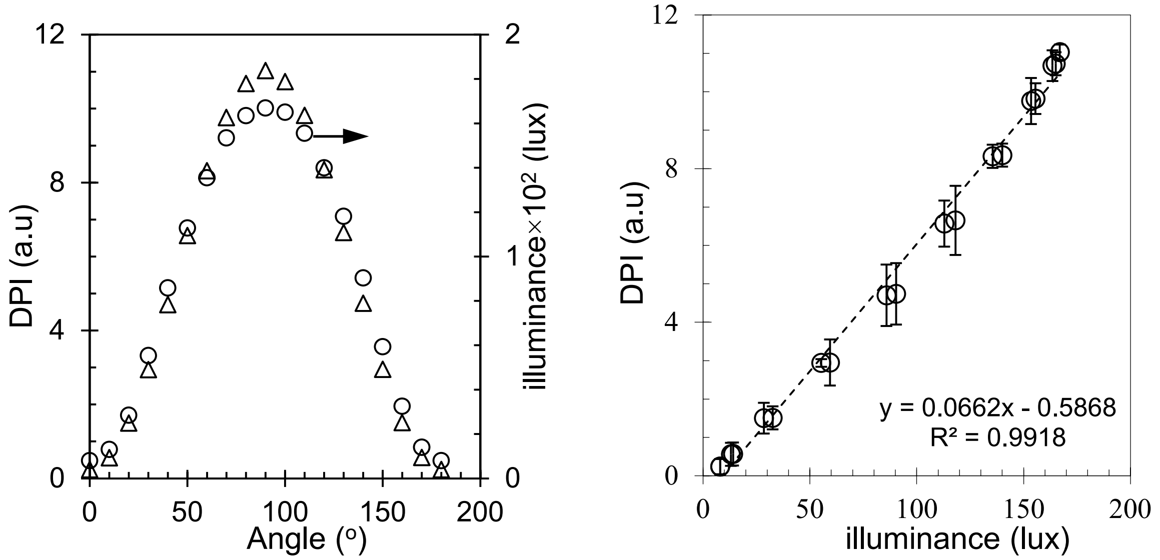

(**a**) (**b**)

Figure S2. (a) Comparison between digitally extracted pixel intensity (DPI), symbolized by triangles, and illuminance, indicated by circles, as they change with rotation angle between the polarizer and analyzer. An arrow highlights the illuminance data plotted on the secondary axis. (b) Plot of DPI as a function of illuminance.

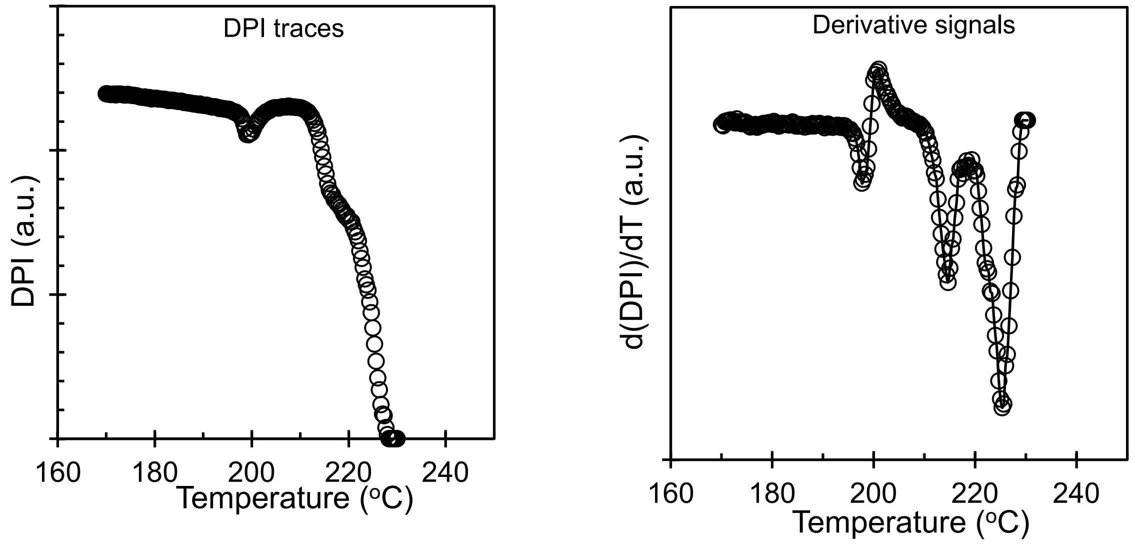

(**a**) (**b**)

Figure S3. (a) Simple variation in DPI traces and (b) derivative of DPI signals with temperature of a polymer sample isothermally crystallized at 170 °C.

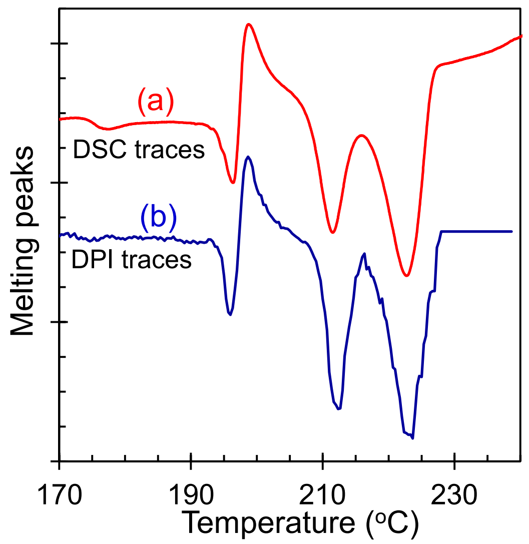

Figure S4. Melting peaks as a function of temperature: (a) conventional DSC (J/g), with endothermic peak oriented downward, and (b) digitally extracted pixel intensity (a.u) for isothermally crystallized iPS held at 170°C for 8h.

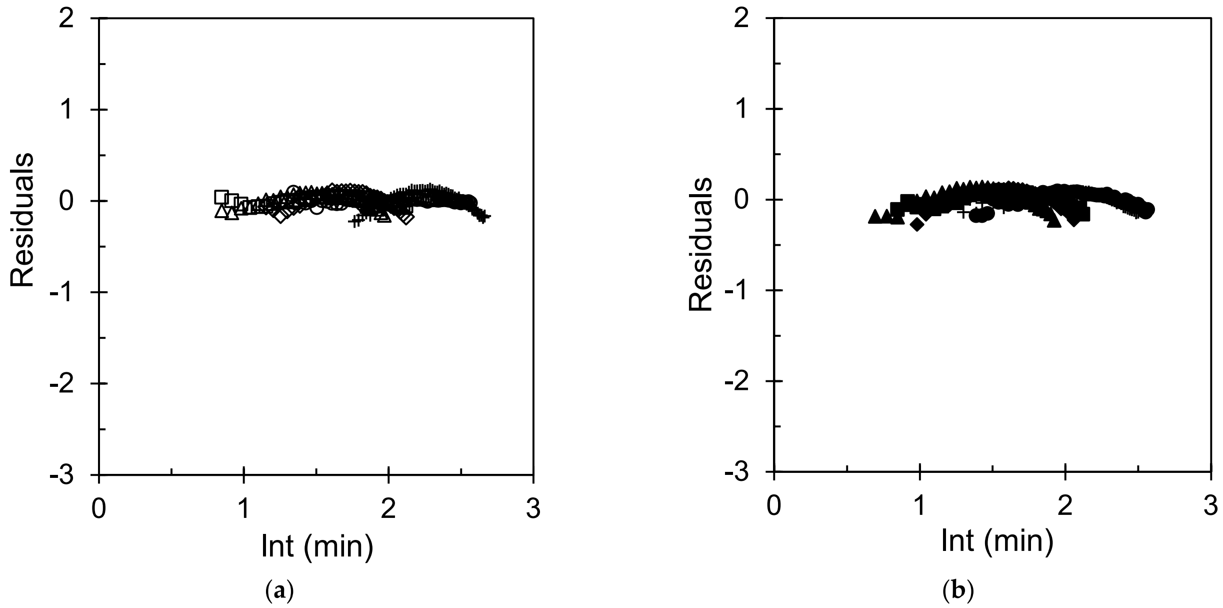

Figure S5. Residuals of the kinetic fits at various crystallization temperatures for samples crystallized from (a) the molten state (open symbols) and (b) the glassy state (solid symbols). The residuals are centered near zero throughout the $\ln t$ (min) range, indicating good fit quality for both crystallization routes. Symbols correspond to the temperature assignments given in Figure 3.

Table S1. Replicate-based statistical summary of kinetic and morphological parameters for Ln*K*, $t_{1/2}^{-1}$ and Log (N_s) across thermal conditions.

Thermal Condition	Parameter	n	Mean	SD	95% CI	p-value	Effect size (η^2)
Melt	Ln*K* (min^{-n})	3	-3.7020	0.500	-4.944069 to -2.459931	7.54E-15	0.979
			-4.3253	0.400	-5.318955 to -3.331645		
			-4.5487	0.400	-5.542355 to -3.555045		
			-6.5556	0.450	-7.673462 to -5.437738		
			-6.7985	0.400	-7.792155 to -5.804845		
			-7.4007	0.400	-8.394355 to -6.407045		
			-9.0932	0.600	-10.583683 to -7.602717		
			-9.9700	0.400	-10.963655 to -8.976345		
			-9.9700	0.300	-10.715241 to -9.224759		
			-9.9900	0.200	-10.486828 to -9.493172		
Glass			-4.3079	0.300	-5.053141 to -3.562659	4.14E-06	0.874
			-5.1179	0.300	-5.863141 to -4.372659		
			-5.8133	0.200	-6.310128 to -5.316472		
			-5.7579	0.400	-6.751555 to -4.764245		
			-6.0528	0.200	-6.549628 to -5.555972		
			-6.0242	0.200	-6.521028 to -5.527372		
			-5.8839	0.300	-6.629141 to -5.138659		
			-6.1547	0.200	-6.651528 to -5.657872		
Melt	$t_{1/2}^{-1}$ (min^{-1})	3	0.2143	0.030	0.139762 to 0.288810	1.05E-10	0.957
			0.1667	0.025	0.104563 to 0.228770		
			0.1111	0.020	0.061428 to 0.160794		
			0.0659	0.018	0.021220 to 0.110649		
			0.0324	0.010	0.007591 to 0.057274		
			0.0165	0.010	-0.008315 to 0.041368		
			0.0105	0.020	-0.039156 to 0.060209		
			0.0105	0.015	-0.026736 to 0.047788		
			0.0105	0.010	-0.014342 to 0.035341		
Glass			0.2222	0.020	0.172539 to 0.271905	1.34E-06	0.891
			0.1736	0.015	0.136300 to 0.210824		
			0.1304	0.010	0.105593 to 0.155276		
			0.1250	0.020	0.075317 to 0.174683		
			0.1250	0.015	0.087738 to 0.162262		
			0.1304	0.010	0.105593 to 0.155276		
			0.1304	0.010	0.105593 to 0.155276		
			0.1250	0.005	0.112579 to 0.137421		
Melt pretreatment temperature of 230 °C	Log N_S (mm^{-3})	3	6.5203	0.012	6.490891 to 6.549730	2.81E-09	0.964
			6.5599	0.012	6.530125 to 6.589759		
			6.5863	0.017	6.544408 to 6.628144		
			6.6044	0.023	6.547553 to 6.661191		
			6.6363	0.018	6.591691 to 6.680832		
			6.6610	0.013	6.629984 to 6.692104		
			6.7324	0.009	6.709394 to 6.755467		
Melt pretreatment temperature of 250 °C			6.5185	0.007	6.501096 to 6.535873	0.992975	0.047
			6.5195	0.010	6.494693 to 6.544376		
			6.5195	0.008	6.499662 to 6.539408		
			6.5195	0.004	6.509598 to 6.529471		
			6.5206	0.003	6.513130 to 6.528035		
			6.5174	0.009	6.495074 to 6.539789		
			6.5216	0.004	6.511691 to 6.531564		
Melt	Log N_S (mm^{-3})	3	6.5542	0.230	5.982887 to 7.125591	6.49E-13	0.983
			6.5061	0.180	6.058995 to 6.953285		
			6.1554	0.080	5.956691 to 6.354153		
			5.4768	0.100	5.228392 to 5.725219		
			4.5119	0.150	4.139315 to 4.884556		
			3.9284	0.180	3.481214 to 4.375504		
			3.6943	0.230	3.122924 to 4.265627		
			3.6273	0.290	2.906929 to 4.347729		
Glass			6.6020	0.210	6.080301 to 7.123639	0.996878	0.055
			6.6366	0.120	6.338520 to 6.934713		
			6.5929	0.180	6.145714 to 7.040003		
			6.5740	0.190	6.102055 to 7.046027		
			6.5959	0.090	6.372344 to 6.819489		
			6.5913	0.120	6.293225 to 6.889418		
			6.6153	0.070	6.441399 to 6.789178		
			6.6503	0.140	6.302495 to 6.998054		
			6.6674	0.160	6.269949 to 7.064873		

Table S2. Avrami fitting parameters corresponding to Figure 4

State of crystallization	T_c (°C)	Fitting range (min)	Conversion window ln (t_c)	Avrami exponent, n	95% CI, n	Rate constant, k (min^{-n})	95% CI, k	Mean residual	R^2
Molten state	150	3.8–13.1	0.157–0.915	2.28±0.28	2.265 to 2.301	0.0072	0.00696 to 0.007511	-1.045E-04	0.999
	160	2.3–8.3	0.114–0.931	2.61±0.32	2.472 to 2.543	0.0109	0.0131 to 0.01473	7.996E-05	0.998
	170	2.3–7.2	0.113–0.936	2.83±0.37	2.747 to 2.928	0.0120	0.01051 to 0.01388	5.370E-05	0.993
	180	3.2–8.3	0.128–0.940	3.24±0.41	3.134 to 3.355	0.0034	0.002852 to 0.004185	3.280E-06	0.992
	190	6.0–14.5	0.127–0.945	3.4±0.29	3.285 to 3.513	0.0004	.0002964 to 0.000502	-9.056E-05	0.986
Glassy state	150	4.1–13.1	0.080–0.967	3.09±0.24	3.024 to 3.143	0.0013	0.001222 to 0.001574	-2.884E-03	0.995
	160	2.3–8.3	0.075–0.969	3.13±0.18	2.949 to 3.082	0.0051	0.006085 to 0.007586	6.640E-05	0.995
	170	2.1–6.8	0.072–0.973	3.19±0.21	3.062 to 3.310	0.0100	0.008264 to 0.0119	1.965E-05	0.99
	180	2.7–8.2	0.114–0.977	3.14±0.23	3.010 to 3.271	0.0074	0.005926 to 0.009098	1.906E-05	0.987
	190	3.7–12.1	0.077–0.972	3.17±0.21	3.110 to 3.236	0.0015	0.001307 to 0.00169	2.104E-05	0.995
